# Sonographic manifestations of multiple medullary carcinoma coexisting with papillary carcinoma of the thyroid: A case report

**DOI:** 10.1016/j.radcr.2025.02.075

**Published:** 2025-03-21

**Authors:** Yuang Xie, Yuxin Zhang, Zhihong Chen, An Wei

**Affiliations:** a Department of Ultrasound, The First Affiliated Hospital of Hunan Normal University (Hunan Provincial People's Hospital), Changsha, China; b Department of Medical Laboratory, The First Affiliated Hospital of Hunan Normal University (Hunan Provincial People's Hospital), Changsha, China

**Keywords:** Medullary thyroid carcinoma (MTC), Papillary thyroid carcinoma (PTC), Ultrasonography, Synchronous malignancy, Lymph node metastasis, TI-RADS

## Abstract

This case report presents a rare coexistence of multiple medullary thyroid carcinoma (MTC) and papillary thyroid carcinoma (PTC) in a 43-year-old woman with a 6-month history of a thyroid mass. Physical examination revealed a firm, irregular isthmus nodule without signs of inflammation. Laboratory tests showed elevated serum calcitonin, carcinoembryonic antigen and gastrin-releasing peptide precursor. Ultrasonography revealed multiple hypoechoic nodules in both thyroid lobes and the isthmus, classified as TI-RADS 4a (right and left lobes), 4b (right lobe), and 3 (isthmus). Postoperative pathology confirmed PTC in the left lobe and MTC in the right lobe and isthmus, both with lymph node metastases. This case underscores the diagnostic challenges of synchronous thyroid malignancies and highlights the key role of high-resolution ultrasound in differentiating pathological subtypes. Comprehensive imaging assessment, combined with biochemical markers and histopathology, is essential for accurate diagnosis and tailored treatment.

## Introduction

Thyroid cancer, the most common endocrine malignancy, predominantly comprises papillary carcinoma (PTC, 80%-85%) and medullary carcinoma (MTC, 1%-2%) [1]. While PTC arises from follicular epithelial cells, MTC originates from parafollicular C cells [2]. The coexistence of these distinct subtypes, termed multiple primary malignancies (MPMs), is exceedingly rare (0.15% of thyroid tumors) [3]. Synchronous MPMs, defined as concurrent tumors diagnosed within 6 months, pose diagnostic challenges due to overlapping imaging features [4]. The diagnosis of MPMs relies on imaging techniques such as ultrasonography (US) and Computed Tomography (CT), which help differentiate primary tumors from metastatic lesions by assessing anatomical, morphological, and vascular features [4,5]. Ultrasonography (US) remains the cornerstone for thyroid evaluation, offering detailed characterization of nodule morphology, vascularity, and calcifications. However, MTC often lacks pathognomonic sonographic features, necessitating adjunctive tools like serum calcitonin and histopathology [5]. The aim of this case was to investigate the diagnostic features of medullary thyroid carcinoma combined with papillary carcinoma by analyzing the ultrasound presentation.

## Case report

A 43-year-old woman was admitted to the hospital with a thyroid lump that had been present for more than half a year. Physical examination: Bilateral neck symmetry, no redness, swelling, or heat pain in the neck skin, and no vascular filling in the neck. The neck was soft, the trachea was centered, the isthmus of the thyroid gland was enlarged to the second degree, and a lump measuring approximately 2.0 cm × 2.2 cm could be seen in the isthmus, and a lump of about 2 cm × 1.2 cm in size could be seen in the isthmus to the right of the isthmus, which was hard, with poorly defined edges, a nonsmooth surface, and no tenderness, and it could move up and down with swallowing but not with tongue extension. The left lobe of the thyroid gland was not obviously enlarged, and no obvious mass was detected. No enlarged lymph nodes were found in the neck bilaterally. Laboratory tests showed elevated levels of carcinoembryonic antigen, gastrin precursor-releasing peptide fragment, and calcitonin; other thyroid function indicators were normal. Ultrasonography: Multiple hypoechoic nodules can be detected in the right lobe of the thyroid gland, with clear borders, irregular morphology, aspect ratio less than 1, and nonuniform internal echoes, one of which is about 1.22 cm × 0.96 cm in size, and multiple strong echogenic spots can be seen in part of it. A hypoechoic nodule was identified in the left thyroid lobe, with clear borders, irregular morphology, aspect ratio less than 1, and uneven internal echogenicity; its size was about 0.56 cm × 0.44 cm, and multiple strong echogenic spots were seen in it. Hypoechoic nodules were detected in the right side of the thyroid isthmus, with clear borders, irregular morphology, aspect ratio less than 1, and nonuniform internal echoes, with a size of about 1.69 cm × 1.02 cm. Hypoechoic nodules could be detected in the cervical IV region bilaterally, with clear borders, regular morphology, and nonuniform internal echoes, with sizes of about 2.12 cm × 0.86 cm (right, multiple) and 0.43 cm × 0.38 cm (left), respectively. Ultrasound suggests: multiple hypoechoic nodules in the right lobe of the thyroid gland, consider TI-RADS category 4a combined with category 4b nonexclusion; hypoechoic nodule in the left lobe of the thyroid gland, consider TI-RADS category 4a; hypoechoic nodule in the isthmus of the thyroid gland, consider TI-RADS category 3; hypoechoic nodules in the IV region of the neck bilaterally, consider abnormal lymph nodes. After completing relevant examinations, enlarged radical thyroid cancer surgery was performed, and pathological diagnosis was made: 1. Papillary carcinoma of the left lobe of the thyroid gland, with a nodule size of about 0.7 cm, adjacent to the peritoneum; cancer metastasis was seen in the lymph nodes of the left central region, left region III, and left region IV. 2. Medullary carcinoma of the right lobe of the thyroid gland, with 2 nodules, with the sizes of 1.8 cm × 1.5 cm × 0.9 cm and 1 cm × 1 cm × 1 cm, respectively, with the larger one adjacent to the peritoneum, and the smaller one infringing on the peritoneum pTNM stage: T1bN1bMx; cancer metastasis was seen in the lymph nodes of the right neck in zones I, III, VIA, and VIB. 3. Medullary carcinoma of the isthmus of the thyroid, size 2 cm × 1.6 cm × 1.3 cm, invading the peritoneum; cancer metastasis was seen in the anterior laryngeal lymph nodes. Immunohistochemistry: CK19 (+), CK7 (+), Ki67 (+), D53 (wild type), TTF-1 (+), CD56 (+), MC (+), BRAF (+), CT (+), TTF-1 (+), p53 (wild type), Syn (+), CgA (+), CD56 (+), INSM1 (+).

## Discussion

Papillary thyroid carcinoma (PTC) and medullary thyroid carcinoma (MTC) have their origins in follicular epithelial cells and parafollicular C cells of the thyroid gland, respectively, and the simultaneous occurrence of the 2 cancers is relatively rare, accounting for only about 0.15% of thyroid tumors. The coexistence of these 2 malignancies is classified as multiple primary malignancies (MPMs), specifically synchronous lesions. MPMs are defined as 2 or more distinct primary tumors occurring in the same patient, either synchronously (within 6 months) or metachronously (after 6 months). This case represents a rare instance of synchronous MTC and PTC, accounting for only 0.15% of thyroid tumors [3]. Imaging techniques, including high-resolution ultrasonography and contrast-enhanced Computed Tomography (CT), play a critical role in detecting MPMs. For example, CT is particularly valuable in staging synchronous tumors due to its ability to evaluate distant metastases [4]. Regarding the mechanism of their occurrence, most scholars are currently in favor of the collision theory, i.e., the simultaneous occurrence of the 2 types of carcinomas is purely coincidental [6]. There is some overlap in ultrasonographic manifestations of MTC and PTC, but due to the different cellular origins of MTC, their ultrasonographic features are specific [4]: MTC is typically located in the middle-to-upper thyroid region and exhibits higher malignancy and invasiveness than PTC, with a richer vascular component of the mesenchyme, larger nodules with a rich blood supply, and is often accompanied by coarse calcification, while PTC is mostly located in the middle portion of the thyroid gland, with relatively small nodules and more often accompanied by coarse calcification. On the other hand, PTCs are usually located in the middle part of the thyroid gland, with relatively small nodules and a lack of blood supply. In conclusion, the ultrasound image of PTC was characterized by being located in the middle portion of the thyroid gland, having an aspect ratio of >1, shallow lobulated margins, solid hypoechoic interior, gravelly calcification foci inside the nodule, and small nodules with a lack of blood supply, while the ultrasound image of MTC was characterized by being located in the middle and upper portion of the thyroid gland, having an aspect ratio of <1, smooth margins, coarse and fine heterogeneous strong echogenicity inside the nodule, larger nodules, and a rich blood supply inside the nodule [7]. Compared with PTC, MTC lacks more typical ultrasonographic manifestations, which makes it difficult to diagnose accurately before surgery, and needs to be combined with calcitonin and other auxiliary diagnostics, and MTC combined with PTC is even rarer in the clinic. In this case, the patient's ultrasound findings showed that the right lobe nodule had typical features of medullary carcinoma, while the left lobe nodule was consistent with the ultrasound manifestation of papillary carcinoma, and the isthmus nodule ultrasound manifestation was atypical, so the ultrasound assessment underestimated the risk level of this nodule. Such different ultrasound features suggest the coexistence of 2 different pathological types and emphasize the need for a thorough assessment of thyroid nodules in clinical practice. This case also shows metastasis to the cervical lymph nodes, which further supports the importance of combining multiple imaging features in ultrasound for comprehensive analysis. The use of high-resolution linear probes is indispensable for thyroid imaging. These probes enhance the detection of subtle features such as microcalcifications and macrocalcifications [8]. Recent advancements in integrated diagnostics, combining US with elastography and contrast-enhanced techniques, further improve diagnostic accuracy for thyroid nodules [8].The coexistence of MTC and PTC in this case underscores the importance of imaging in diagnosing and staging MPMs. Contrast-enhanced CT is pivotal for detecting synchronous tumors in deep-seated organs (e.g., lungs, liver) and assessing metastatic lymph nodes during initial staging [4]. In a study by Kowalczyk et al. [4], CT identified synchronous malignancies in 8.2% of cancer patients, emphasizing its role in comprehensive staging. For thyroid malignancies, US remains the first-line modality, but integrating CT provides a holistic view of extrathyroidal spread and distant metastases, particularly in aggressive subtypes like MTC.

## Conclusion

**Fig. 1:**
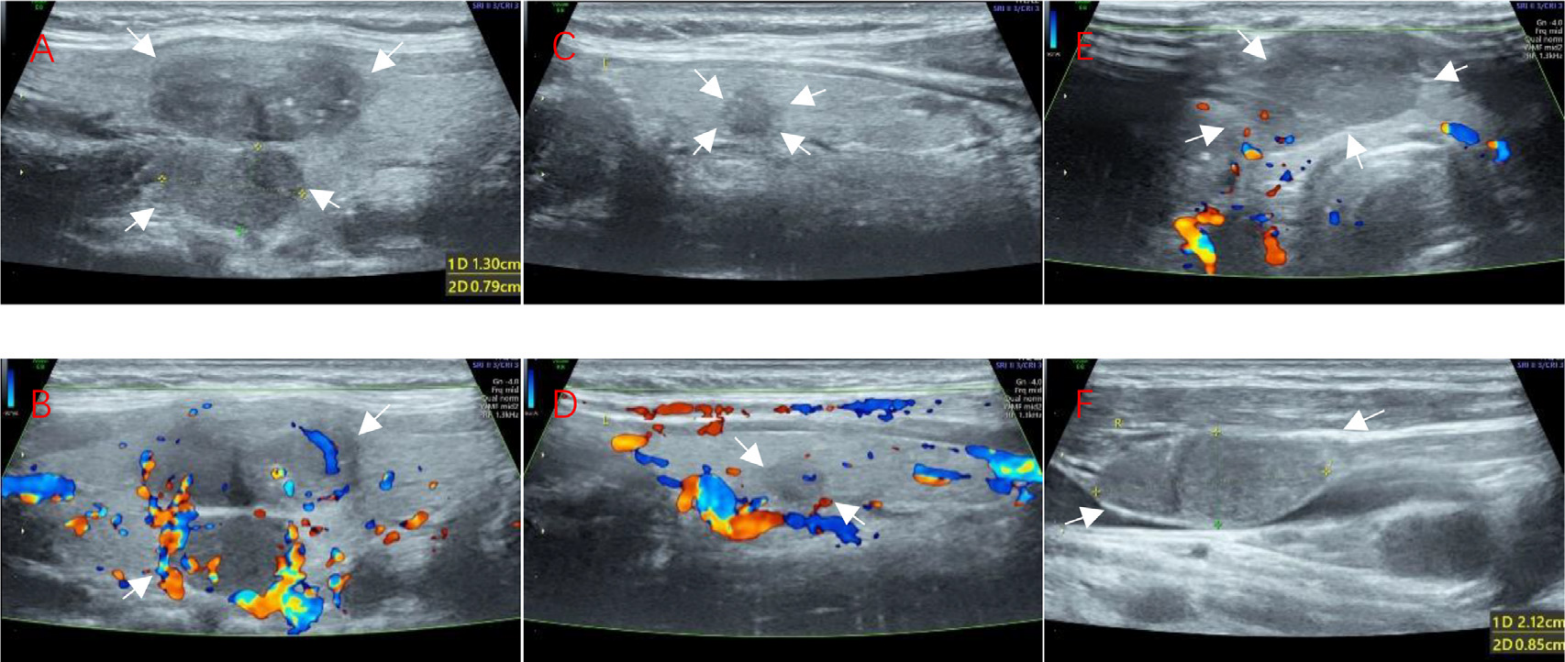
Ultrasonograms of Nodules in Both Lobes of the Thyroid Gland (Arrows Show Nodules) (Ultrasonic diagram obtained with a 21 MHz model 11L-D linear transducer from a Voluson E8.) (A) 2D ultrasonogram of multiple nodules in the right lobe; (B) Color ultrasonogram of multiple nodules in the right lobe; (C) 2D ultrasonogram of nodules in the left lobe; (D) Color ultrasonogram of nodules in the left lobe; (E) Color ultrasonogram of isthmus nodules; (F) Right cervical region VI lymph nodes.

**Fig. 2:**
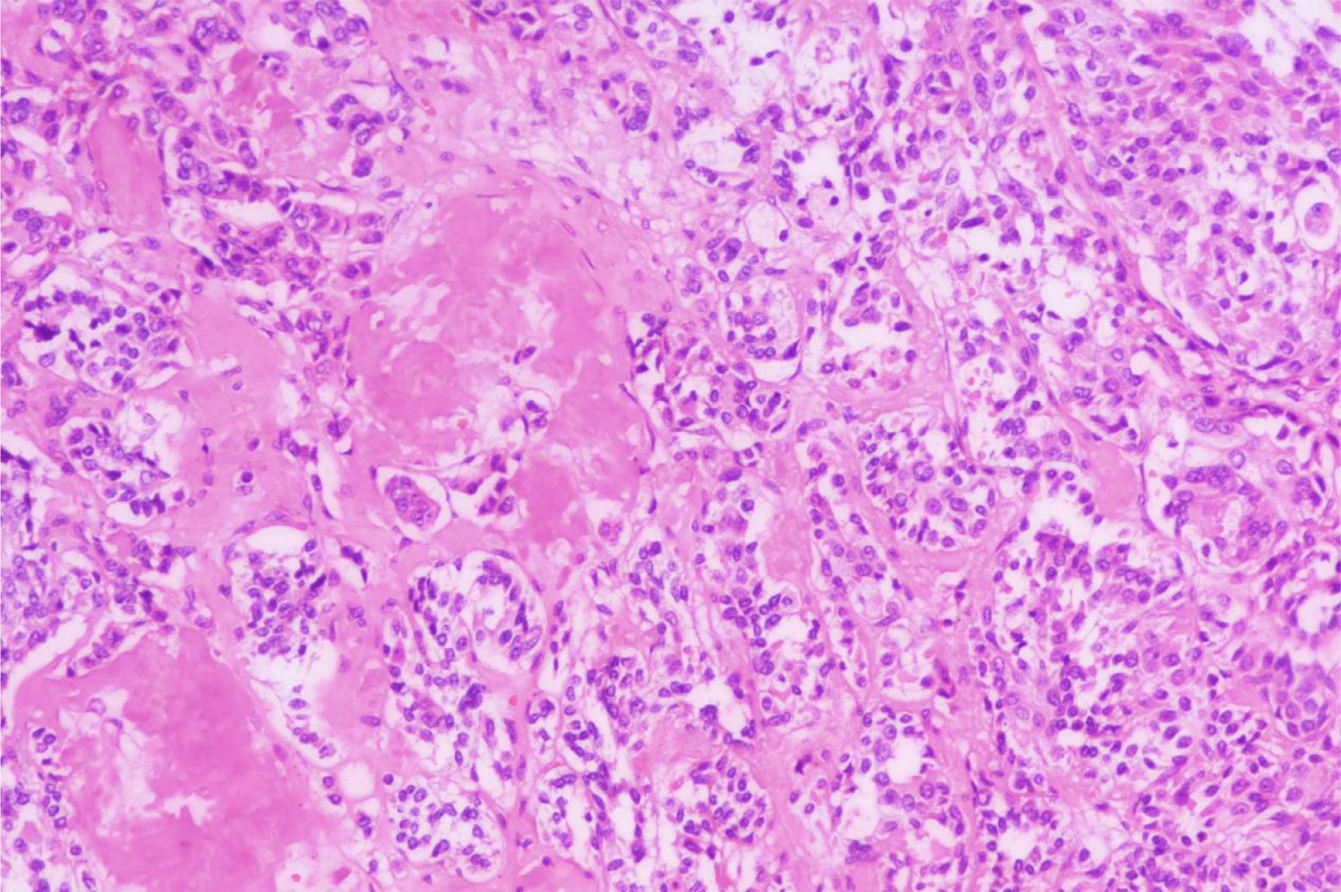
Histopathological sections of medullary thyroid carcinoma (HE, X200).

This case highlights the importance of ultrasonography in the diagnosis of thyroid cancer, especially when different pathological types coexist. Meticulous ultrasound evaluation and pathological analysis can lead to more accurate diagnosis and treatment of thyroid cancer and improve patient survival and quality of life. Therefore, in clinical work, if nodules in the same gland are found to exhibit different ultrasound image features and bilateral neck lymph node metastases (LNMs) also have different ultrasound image features, it is necessary to be alert to the coexistence of thyroid cancers of different pathological types, and it is recommended to perform biopsy or other related examinations separately to clarify the diagnosis at an early stage, which will help the clinic to choose the appropriate treatment modality (Figs. 1 and 2).

## Author contributions

All authors contributed to the conception, design, and drafting of this submission in its final format.

## Patient consent

Written informed consent for the publication of this case report was obtained from the patient.

## Declaration of Competing Interest

The authors declare that they have no known competing financial interests or personal relationships that could have appeared to influence the work reported in this paper.
